# Obesity and psychological distress

**DOI:** 10.1098/rstb.2022.0225

**Published:** 2023-10-23

**Authors:** Andrew Steptoe, Philipp Frank

**Affiliations:** ^1^ Department of Behavioural Science and Health, University College London, 1-19 Torrington Place, London WC1E BT, UK; ^2^ Department of Epidemiology and Public Health, University College London, 1-19 Torrington Place, London WC1E BT, UK

**Keywords:** adiposity, depression, symptomatology, inflammation, stigma, discrimination

## Abstract

The relationship between high body weight and mental health has been studied for several decades. Improvements in the quality of epidemiological, mechanistic and psychological research have brought greater consistency to our understanding of the links. Large-scale population-based epidemiological research has established that high body weight is associated with poorer mental health, particularly depression and subclinical depressive symptoms. There is some evidence for bidirectional relationships, but the most convincing findings are that greater body weight leads to psychological distress rather than the reverse. Particular symptoms of depression and distress may be specifically related to greater body weight. The psychological stress induced by weight stigma and discrimination contributes to psychological distress, and may in turn handicap efforts at weight control. Heightened systemic inflammation and dysregulation of the hypothalamic-pituitary-adrenal axis are biological mechanisms that mediate in part the relationship of greater body weight with poorer mental health. Changing negative societal attitudes to high body weights would improve the wellbeing of people living with obesity, and promote more effective weight-inclusive attitudes and behaviours in society at large, particularly in healthcare settings.

This article is part of a discussion meeting issue ‘Causes of obesity: theories, conjectures and evidence (Part II)’.

## 1. Introduction

For much of human history, a large body size and heavier weight has been regarded as a symbol of affluence, prosperity and high social status, making it likely that obesity was associated with good mental and physical health [1]. However, as the negative consequences of obesity were increasingly understood in the twentieth century, high body weight came to be conceptualized as a psychopathological condition and the province of mental health specialists. This link was amplified by the development of research on eating disorders such as bulimia nervosa and binge eating disorder that shows strong associations with depression, social anxiety and distress [2], particularly because some risk factors for eating disorders such as negative body image are also linked with obesity [3]. These clinical perspectives have been complemented by research which recognizes that people living with obesity who attend mental health clinics are not necessarily typical, and that understanding the relationship between obesity and mental health requires larger-scale systematic work based on the general population. The results of this approach have not been entirely clear-cut, because while some studies find that obesity is associated with depression and anxiety [4], others argued that people living with obesity have notably good psychological wellbeing, coining the unfortunate term ‘jolly fat’ [5].

This article provides a contemporary overview of the relationship between high body weight and mental health. The focus is on the spectrum of psychological distress ranging from negative mood states to severe depressive symptoms and major depressive disorder (MDD), because this is the domain for which the evidence is most compelling. Space prevents discussion of broader issues of mental health and body weight in children and adolescents, or weight gain and mood in pregnancy.

We address five main issues. First, we discuss whether the association between high body weight and psychological distress is positive or negative. We will argue that the overwhelming evidence is that greater body weight is correlated with heightened distress and depressive symptoms in high-income countries in the present day, although the pattern may be different historically and in lower income countries. Second, we consider the question of the direction of association: is psychological distress a risk factor for higher body weight, does higher weight status lead to increased distress, or are there reciprocal associations? We conclude that the majority of findings are consistent with the conclusion that higher body weight leads to increased distress, although distress associated with living with overweight and obesity may subsequently help maintain greater body weight. Third, we consider whether there are specific symptoms of depression and negative emotional states that characterize high body weight. Fourth, we then outline the mechanisms that link high body weight with psychological distress, addressing both emotional factors such as perceived stigma and discrimination, and biological pathways including the hypothalamic-pituitary-adrenocortical (HPA) axis, and systemic inflammation. Finally, we touch on the implications of research on psychological distress and higher weight status for policies promoting healthy weight and the better management and societal treatment of people who are living with overweight or obesity.

## 2. Is high body weight associated with good or poor psychological wellbeing?

An influential study by Crisp & McGuiness published in the *British Medical Journal* in 1976 argued that obesity is coupled with good mental health [5]. This work involved a survey of 739 men and women aged 40–65 recruited from a general practice in southwest London. Obesity was not assessed in a standard fashion, while the measure of mental health (the Middlesex Health Questionnaire) has been little used in recent decades. A very small proportion of participants (5% of men and 3.3% of women) were classified as living with obesity. Men living with obesity reported lower anxiety and depression than those with healthy weight, while differences among women were significant for anxiety but not depression. There was no adjustment of sociodemographic factors or other health problems. Nonetheless, the study popularized the notion that living with obesity is associated with being ‘jolly’ and having good mental health.

This has not, however, been borne out by more robust studies that provide strong support for greater body weight being related to poor mental health. The most compelling evidence comes from meta-analytic reviews of large-scale population-based and case-control studies. These investigations have typically ascertained data on adiposity from measures of height and weight to compute body mass index (BMI), although other measures of body fat composition have been used, such as waist circumference or waist-hip ratio. To date, six meta-analyses have confirmed a cross-sectional link of obesity (BMI ≥ 30 kg m^−2^ or abdominal obesity) with depressive symptoms or clinical depression in adult populations, reporting minimally adjusted pooled risk ratios ranging between 1.18 and 1.42 [6–12]. These meta-analyses have synthesized data from more than 80 studies from North America, Europe and Asia, with sample sizes ranging from 12 292 to 371 897 individuals. Sex has been found to moderate the cross-sectional relationship between obesity and depression, with stronger associations observed in women compared with men [12,13]. The underlying reasons for these sex differences remain uncertain. However, observational data suggest that women encounter weight bias more frequently, amplifying the risk of mental health issues such as depression and anxiety (see §4a) [14]. Menopause-related hormonal changes could provide an additional explanation, as they have been associated with both weight gain and depression [15,16]. Evidence on the relationship of overweight with depression is less convincing, with some studies showing modest, and others null associations [9,10,12].

Notwithstanding the strength of this evidence, there continue to be findings showing positive associations between living with obesity and good mental health. For example, a recent meta-analysis of people aged 60 and over concluded that individuals living with overweight and obesity were less likely to have major depression than those in the normal weight range, and were likely to have fewer symptoms of depression as well [11]. It is notable that of the 14 studies included in this meta-analysis, 12 were carried out in Asian countries such as Korea, China and Taiwan. This raises the possibility that cultural factors in the valuation of high body weight may operate in some societies, particularly among older people with limited education [17]. For example, there is a traditional saying ‘laugh and grow fat’ in China that may continue to be lived out among older generations [18].

## 3. Longitudinal studies of associations between body weight and psychological distress

**Figure 1.**
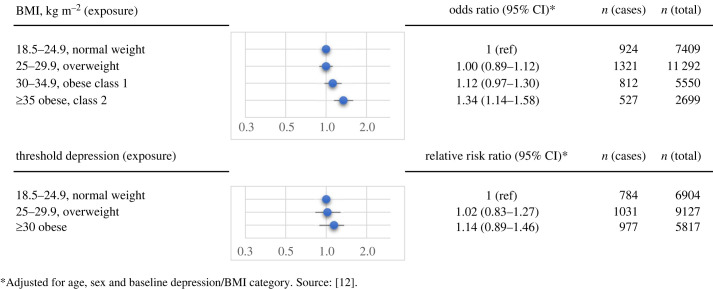
Longitudinal bidirectional associations between BMI and depression.

Longitudinal studies provide information about the direction of these associations. They can assess the development of future poor mental health among individuals living with obesity at baseline, and conversely the progression towards greater body weight among people suffering from depression and distress at baseline. Several longitudinal population studies have been conducted, and two meta-analytic investigations of longitudinal data supported robust bidirectional links. It appears that higher weight status increases the risk of depression, while depression also predicts future high body weight [13,19]. Specifically, individuals living with obesity had 18–55% increased odds of developing depression, whereas the risk of being classified as having obesity in people with depression was increased by 37–58%. By contrast, our more recent individual-level meta-analysis of population-based data found no evidence for an elevated risk of having obesity in individuals with depression as ascertained from validated self-report measures [12]. Figure 1 provides a summary of our findings, showing that increasing levels of BMI at baseline are associated with progressively increased risk of depression in the future over a mean follow-up period of 3.2 years (upper panel). By contrast, there was no statistically significant relationship between depression at baseline and subsequently being classified as obese (lower panel). Several limitations in our study may account for the lack of association between baseline depression and later higher body weight. Specifically, we were unable to differentiate obesity into its clinically relevant classes or consider varying severity levels of depression in longitudinal analyses investigating the risk of high body weight following depression. Indeed, in a more recent multicohort study of over 240 000 individuals from the United Kingdom (UK) and Finland, we found that individuals with moderately severe to severe depression had an almost sevenfold higher risk of being hospitalized owing to obesity during a 5-year follow-up compared with those without depression [20]. It is also worth noting that the association of obesity with depression appears to be stronger in studies using manual and clinically-based measures of MDD (e.g. clinical interviews) compared with those relying on self-report measures of depressive symptoms. Accordingly, evidence from a previous meta-analysis synthesizing the data from 15 longitudinal studies comprising a total of 58 745 participants suggests that being classified as obese is associated with 2.15-fold (95% confidence intervals (CI) 1.48–3.12) increased odds of subsequent clinical depression, whereas the odds of self-reported depression were only 1.36 (1.03–1.80). Conversely, the odds of developing obesity were 1.71 (1.33–2.19) in people with clinical depression, and 1.48 (1.17–1.87) in those with self-reported depression ascertained from validated self-report measures [13].

Overall, research suggests that the obesity-depression link is largely robust to multivariable adjustment for socio-economic and lifestyle-related factors [13,19], and follows a dose-response pattern, with stronger associations evident in people living with more severe obesity [21]. However, the long-term effects of weight change on mental health across the life course remain unclear, and there is a dearth of studies that have examined the association of weight change with depression over time. Preliminary evidence from the Tasmanian Longitudinal Health Study involving 2416 participants suggests that weight gain from childhood to mid-adulthood is associated with a higher risk of adulthood depression and greater symptom severity [22]. While the long-term effects of weight gain on mental health require further testing in larger population-based studies, there is also a need for future research to examine the effects of intentional weight loss on psychological wellbeing. To date, only one clinical trial has examined long-term changes in depression following a 12- or 52-week commercial behavioural weight management programme, revealing no significant differences between the intervention and control group during a 5-year follow-up period [23].

### (a) High body weight and specific symptoms of depression and distress

It is important to note that depression is a heterogeneous disorder with varying types of symptom expressions, and that symptoms of depression are not pathognomonic (i.e. disease-specific) [24]. The diagnostic classification of depression is embodied in the diagnostic statistical manual (DSM) of Mental Health Disorder 5th version [25], and the International Classification System of Diseases 11th edition [26]. These taxonomies lay out a set of criteria for defining depression, including symptom specifiers, time course, and background circumstances. Per DSM-5, a diagnosis of MDD necessitates at least one of two key symptoms—low mood or anhedonia, plus four or more other symptoms including appetite changes, sleep issues, fatigue, concentration problems and suicidal ideation. By contrast, the International Classification of Diseases, ICD-11 lists a total of three cardinal symptoms—depressed mood, anhedonia, loss of energy, with at least two core symptoms and two additional symptoms required for a depression diagnosis.

In recent years, new research endeavours have emerged to examine whether obesity is differentially associated with specific symptom patterns rather than depression more generally. Initial studies in this field have focused on two specific symptom features within MDD: melancholic and atypical depression. Accumulating evidence from large-scale case-control studies suggests that obesity and obesity-related immunometabolic dysfunction may be more common in patients exhibiting an atypical symptom profile [27,28]. However, a comparison of results across these studies is challenging by differences in the operationalization of atypical depression [21]. Moreover, thus far, there has been no consensus in the scientific literature on the usefulness of depression subtypes [29–32], mainly owing to their limited discriminant validity and the absence of better treatment outcomes in anti-depressant trials stratifying patient samples according to depression subtypes.

It has therefore been argued that a more granular symptom-level approach is needed to better understand the link between biological risk factors such as high body weight and depression [33]. Indeed, three lines of evidence point towards symptom-specific associations of excess body weight with depression that, in combination, are not attributable to any existing diagnostic categories: case-control studies of clinical populations [34,35], observational studies of general populations [36], and studies using genetically informed designs [37,38]. These studies have typically relied on self-report measures that assessed symptoms within and beyond the diagnostic criteria of depression. Data from previous case-control and population studies have demonstrated that high body weight and obesity-related leptin dysfunction were primarily related to somatic symptoms such as reduced energy levels, fatigue, and sleep problems, but also to a subset of emotional and self-perception-related symptoms such as feelings of inadequacy and anhedonia. However, these studies are generally characterized by relatively small sample sizes, used cross-sectional study designs, and did not include subgroup analyses to test the robustness of associations across multiple cohorts from different countries.

We therefore carried out a large-scale investigation that pooled individual-level data from 15 population-based cohort studies from the UK, Ireland, USA, Mexico and other countries, in addition analysing UK Biobank data in a replication analysis [12]. We examined the cross-sectional and longitudinal associations of overweight, obesity class I, and obesity classes II-III with overall depression status and 24 individual symptoms of depression in up to 180 000 adults. The results revealed that, in cross-sectional analyses, obesity status, but not overweight, was robustly associated with *lack of energy*, the anhedonia-related symptom *little interest in doing things*, *feelings of inadequacy* and *feeling depressed*. Notably, individuals living with obesity were two times more likely to experience three or four of these symptoms than to experience overall depression. We also observed that symptom-specific associations were stronger in more severe obesity classes and in women compared with men. Furthermore, obesity preceded rather than followed the development of these symptoms in longitudinal analysis.

A third line of evidence for symptom specificity comes from genetic studies. To date, two Mendelian randomization (MR) studies have examined the association between genetic variants related to increased BMI and individual depressive symptoms [37,38]. The first MR study found that genetically upregulated BMI was associated with four of the nine depressive symptoms listed the Patient Health Questionnaire, including *tiredness*, *anhedonia*, *feelings of inadequacy* and *changes in appetite*. By contrast, no association was evident with a clinical diagnosis of MDD [37]. It is worth noting that the associations identified in this study are strikingly similar to those observed in our individual-level meta-analysis [12]. The second investigation aimed to further examine whether the association with changes in appetite was characterized by increased or decreased appetite, and found that, perhaps unsurprisingly, genetically elevated BMI was associated with hyperphagia [38]. While symptom specificity in the association of high body weight with depression or, more generally, psychological distress, requires further testing, future research is needed to explore whether these symptoms are a direct somatic consequence of excess body weight or represent independent mood symptoms, and whether individuals exhibiting such symptoms may benefit from conventional anti-depressant treatments.

## 4. Mechanisms linking high body weight with psychological distress

There are multiple ways in which high BMI is linked with psychological distress, but here we focus on two broad sets of processes through which high body weight may stimulate the development of depression or exacerbate existing mental health issues: behavioural/emotional factors, and biological pathways involving HPA dysregulation and inflammation. Prominent among the behavioural and emotional factors are the adverse experiences of people with high body weight resulting from negative interpersonal and social attitudes.

### (a) Weight stigma and discrimination

One reason why many people living with obesity experience severe psychological distress is because they are exposed to weight stigma and discrimination. These can be defined as negative societal perceptions of overweight and obesity. People with greater body weight are frequently devalued and undermined, being characterized as lazy, lacking in self-discipline, and weak. Discrimination is apparent in multiple settings from employment and education to health care, with evidence that people with higher body weight are less likely to be promoted, are regarded as less academically able so ignored in educational settings, and less worthy of treatments for other health conditions [39]. Stigma in educational settings is particularly damaging because it can lead to school avoidance and low academic achievement, resulting in downward socio-economic trajectories as opportunities for advancement are lost. It emerges in teasing and bullying by peers as well as devaluation from teachers, and in recent years online bullying has become an increasing problem [40]. These tropes are reinforced in portrayals in the mass media, including TV, shops, newspapers and magazines, social media and even children's books, where characters living with overweight and obesity are portrayed as unpleasant, stupid, or figures of fun. Women living with overweight are particularly targeted [41].

The prevalence of stigma has been challenging to estimate owing to the limited scope of many studies, which often rely on small, selectively chosen samples or analyses of individuals participating in commercial weight loss programmes. However, an analysis of the Midlife in the United States Study found that some 40% of adults with a BMI of 35 and above reported some form of discrimination [42]. A more recent nationally representative sample of men and women aged 50 and older in England showed that 24.2% of people categorized as class II and 35.1% of class III experienced discrimination on the basis of their weight [43]. Experiences of stigma and discrimination can become internalized, with individuals of high body weight seeing themselves as to blame for their situation, enhancing body image problems, and undermining self-confidence and self-esteem.

One consequence may be overeating and the consumption of calorific comfort foods, further increasing body weight [44]. Another is increased psychological distress. A meta-analysis of 105 cross-sectional studies with multiple mental health outcomes showed associations between perceived weight stigma and greater depression, anxiety, psychological distress and poor quality of life [45], all of the reviewed studies were cross-sectional so longitudinal relationships could not be established, and although psychological outcomes were worse for people of higher body weight, there were no associations with sex or age. Research on weight stigma and psychological distress has focused on the perceptions of victims rather than on objective indicators of discrimination. This is not surprising because the fact that an observer regards certain situations as discriminatory does not mean that the individual appraises these situations in the same way, and many people with high body weight learn to cope effectively [46]. How important weight stigma is to weight maintenance and weight gain is difficult to gauge; it is notable that studies of weight stigma almost always taken BMI into account, but that studies of mechanisms related to weight gain rarely control for weight stigma.

### (b) Hypothalamic-pituitary-adrenocortical axis dysregulation

A key biological correlate and predictor of both depression and obesity is HPA-axis dysregulation. The HPA-axis is primarily known in the mental health field for its role as one of the body's most effective stress-response systems as it involves a variety of neuroendocrine pathways that regulate an array of stress-induced physiological processes associated with the immune system, metabolism, and the autonomic nervous system. In response to stress, the hypothalamus secretes the corticotropin-releasing hormone (CRH), which then stimulates the release of adrenocorticotropic hormone which in turn promotes the secretion of glucocorticoids such as the steroid hormone cortisol from the adrenal cortex. Glucocorticoids provide negative feedback to the hypothalamus to regulate the stress-response (i.e. inhibition of CRH production) and maintain homeostasis. Glucocorticoid receptors exist throughout the body, including the brain. Repeated activation of the HPA-axis has been linked to reduced sensitivity of glucocorticoid receptors; a condition known as glucocorticoid resistance. Glucocorticoid resistance prevents cortisol from providing adequate negative feedback to the hypothalamus, which can result in a chronic stress-response characterized by the continued production and secretion of stress hormones. Importantly, glucocorticoid resistance is one of the most replicated findings in patients with MDD, and has also been related to impaired cognitive functioning and a poorer treatment response to existing antidepressant therapies [47,48]. Meta-analytic evidence synthesizing data from 20 case-control studies has confirmed altered HPA-axis activity in MDD patients as indexed by higher levels of cortisol [49]. In addition, elevated morning cortisol has been found to predict future depression [50,51]. Unfortunately, despite the strength of the current evidence on the role of the HPA-axis in depression, intervention studies on pharmacological treatments targeting HPA-axis function (e.g. glucocorticoid receptor antagonist) have yielded mixed or null results [52–54].

HPA-axis hyperactivity indexed by excessive amounts of cortisol has also been observed in people living with obesity. In fact, it has been estimated that almost 50% of adults with BMIs categorized as obese also show excessive levels of cortisol [55], and that obesity is associated with dysregulation of HPA stress reactivity [56]. Furthermore, there is evidence that systemic glucocorticoid therapy used to treat patients with asthma or chronic obstructive pulmonary disease is often accompanied by weight gain, suggesting that glucocorticoid dysregulation may contribute to the development of high body weight [57]. Interestingly, individuals living with obesity who report weight discrimination have 33% higher cortisol concentrations than those who do not, with mediation analysis indicating that discrimination on the basis of weight partly accounts for the links between greater adiposity and cortisol output [58].

### (c) Systemic inflammation

A further biological mechanism linking excess body weight with depression involves disturbances of the innate immune system, in particular systemic inflammation. Inflammation refers to a set of immune-related processes that are activated in response to endogenous and exogenous challenges to the body. The primary purpose of inflammation is to maintain homeostasis, which includes, for example, the elimination of harmful stimuli such as pathogens, as well as the disposal and repairing of damaged cells and tissue. As such, inflammation is an adaptive biological response that promotes healing. However, a dysregulated innate immune response may result in a prolonged inflammatory state, which can be both cause and consequence of severe medical conditions such as arthritis, coronary heart disease, and type-2 diabetes [59–61].

It is well known that systemic inflammation is associated with high body weight [62]. Key proinflammatory markers such as interleukin 6 (IL-6) and the acute phase reactant C-reactive protein (CRP) are synthesized by white adipocytes after macrophage infiltration [63], and higher levels of body fat, in particular visceral fat, are robustly associated with metabolic inflammation [21,64,65]. Increased levels of CRP in people with high body weight have been previously confirmed in a meta-analysis of 51 cross-sectional population-based studies [66]. There is also a strong case for supposing that inflammation plays a role in psychological distress. In rodent models, peripheral inflammation stimulated by endotoxins induces depression-like behaviours [67]. In human studies, the administration of proinflammatory agents, such as interferon-alpha or IL-2, has been shown to induce depressive symptoms in some 40% of patients with prior immune-related physical illnesses, such as hepatitis C and cancer [68–70]. Furthermore, meta-analytic reviews of the potential antidepressant role of immunoregulatory drugs in inflammation-related diseases suggest small to moderate effects of non-steroidal anti-inflammatory drugs and anti-cytokine-based drugs in alleviating depressive symptoms [71–74]. In addition to clinical trial data, several meta-analytic reviews of clinical case-control and population-based cohort studies have described elevated levels of circulating proinflammatory cytokines, acute-phase reactants and chemokines in people with depressive symptoms or MDD [75–81].

Little research has examined the mediating role of inflammation in the body weight-depression link. While, in our recent individual participant meta-analysis, we were not able to test mediation formally, we calculated the percentage of attenuation of the high body weight-depression association following adjustment for CRP and a history of or current obesity-related diseases in a subsample of 83 385 participants from the UK Biobank study [12]. The results in table 1 indicate that 18.96% of the relationship between high body weight and depression was attributable to systemic inflammation in models adjusted for age and sex, suggesting a potential mediating role of CRP. Furthermore, a recent study of a nationally-representative sample of men and women aged 50 and older in England found that CRP acted as a partial mediator of the association between excess body weight and elevated somatic, but not cognitive-affective, or overall depressive symptoms, explaining a total of 15% of the association [82].

**Table 1.** Percentage attenuation of the association between BMI and depression following adjustment for C-reactive protein. (ref, reference category.)

BMI, kg m^−2^ (exposure)	depression/depressive symptoms (outcome)	odds ratio (95% CI)								
		model 1^a^	model 2^b^	attenuation (%)	model 3^c^	attenuation (%)	model 4^d^	attenuation (%)	*n* (cases)	*n* (total)
18.5–24.9, normal weight	depression (overall)	1 (ref)	1 (ref)		1 (ref)		1 (ref)		2347	55 093
> 30, obese		2.72 (2.56 to 2.88)	2.25 (2.11 to 2.40)	18.96	2.51 (2.37 to 2.67)	8.03	2.11 (1.98 to 2.25)	25.38	2762	28 292
18.5–24.9, normal weight	lack of energy	1 (ref)	1 (ref)		1 (ref)		1 (ref)		4804	55 093
> 30, obese		2.27 (2.17 to 2.37)	1.89 (1.80 to 1.99)	22.35	2.09 (2.00 to 2.19)	10.08	1.77 (1.69 to 1.86)	30.35	4723	28 292
18.5–24.9, normal weight	little interest in doing things	1 (ref)	1 (ref)		1 (ref)		1 (ref)		1617	55 093
> 30, obese		2.23 (2.08 to 2.39)	1.87 (1.73 to 2.03)	21.95	2.09 (1.94 to 2.24)	8.08	1.77 (1.64 to 1.92)	28.81	1697	28 292
18.5–24.9, normal weight	feeling bad about myself	1 (ref)	1 (ref)		1 (ref)		1 (ref)		1897	55 093
> 30, obese		2.11 (1.97 to 2.25)	1.86 (1.73 to 2.00)	16.89	1.99 (1.86 to 2.13)	7.84	1.77 (1.65 to 1.91)	23.53	1794	28 292
18.5–24.9, normal weight	feeling depressed	1 (ref)	1 (ref)		1 (ref)		1 (ref)		1511	55 093
> 30, obese		2.07 (1.92 to 2.23)	1.74 (1.60 to 1.89)	23.87	1.94 (1.80 to 2.09)	8.91	1.65 (1.52 to 1.79)	31.17	1453	28 292

^a^Odds ratios adjusted for age and sex.

^b^Odds ratios adjusted for age, sex and C-reactive protein level at baseline.

^c^Odds ratios adjusted for age, sex and history of/current at least one of 21 common obesity-related diseases at baseline.

^d^Odds ratios adjusted for age, sex, C-reactive protein, and current or a history of at least one of 21 common obesity-related diseases at baseline.

## 5. Implications for the support of people living with high body weight

Studies of high body weight and psychological distress not only throw light on the aetiology and maintenance of these problems, but also have implications for the support of individuals living with overweight or obesity. The research described in earlier sections demonstrates the close links between negative attitudes to high body weight, poor mental health, and the biological underpinnings of high body weight. It has sometimes been argued (implicitly or even explicitly) that negative attitudes to adiposity will somehow shame individuals with high body weight into making greater efforts to regulate food intake and increase physical activity, so should be encouraged. However, any such marginal benefit of ‘fat shaming’ must be set against the strong evidence for adverse effects on psychological wellbeing. Far from promoting weight loss, such attitudes may lead to weight gain. So an important implication of this research is to discredit any value that clinicians, the media, and the public place on fat shaming, by highlighting the negative links between living with obesity and mental health.

Many people with high body weight cope with issues of body dissatisfaction by maintaining healthy lifestyles that can in turn reduce psychological distress. This may include exercise regimens and other lifestyle modifications that prioritize health over concern about body weight. Acceptance-based coping responses such as not worrying about what other people think, or reducing the importance of this aspect of appearance on self-image, may also reduce depression and distress [46]. The new generation of glucagon-like peptide-1 analogue medications not only lead to substantial weight loss, but may also improve quality of life including psychological wellbeing [83,84]. It remains to be seen whether levels of severe depressive symptoms are reduced. However, it is notable that the monitoring of psychological wellbeing typically has a peripheral role in weight management policies, and greater attention to the mental health correlates and consequences of weight management interventions would probably improve the quality of life of individuals with high body weight.

Societal prejudice is difficult to shift because it is so embedded in contemporary culture. National and international scientific organizations involved in obesity research are becoming more vocal in their opposition to fat shaming (e.g. https://aso.org.uk/news/joint-statement-response-times-article-fat-shaming-only-way-beat-obesity-crisis). However, one place to start promoting change is in healthcare settings, where stigma is rife [85]. Although prejudice can be explicit in the form of derogatory comments and insensitive language, much of the problem is implicit, apparent in subtle signs such as limited eye contact and touch, patronizing interactions, and attribution of medical problems to the person's weight [86]. Systematic proposals for reducing weight stigma are now being put forward. For example, Talumaa *et al*. [85] have reviewed a range of studies testing programmes focused on reducing weight stigma in healthcare, identifying several promising strategies. These include educational programmes that highlight the issues, weight-inclusive health promotion strategies, and greater efforts to understand lived experience. Success in changing weight biases and stigma has been variable, but a more comprehensive approach that integrates mental health with our understanding of the biological determinants of severe adiposity may prove valuable.

## Data Availability

This article has no additional data.
